# Contributions of *NR1H3* genetic polymorphisms to susceptibility and effects of narrowband UVB phototherapy to nonsegmental vitiligo

**DOI:** 10.1038/s41598-023-30047-7

**Published:** 2023-02-28

**Authors:** Meifeng Xu, Qiuyu Xu, Yan Liu, Xiaoli Li, Mei Wang, Wei Dong, Yuning Song, Shengxiang Xiao

**Affiliations:** 1 Department of Dermatology, The Second Affiliated Hospital of Xi’an Jiaotong University, 157 Xiwu Road, Xincheng District, Xi’an, 710004 Shaanxi China; 2 School of Medicine and Forensics, Xi’an Jiaotong University Health Science Center, Xi’an, Shaanxi China; 3 Department of Laboratory Medicine, The Second Affiliated Hospital of Xi’an Jiaotong University, Xi’an, Shaanxi China; 4 School of Life Science and Technology, Xi’an Jiaotong University, Xi’an, Shaanxi China

**Keywords:** Risk factors, Genetic markers

## Abstract

Vitiligo is the most common depigmenting disorder to which both genetic and environmental factors contribute. The aim of the current work was to evaluate the relationship between polymorphisms of the gene nuclear receptor subfamily 1 Group H member 3 (*NR1H3*) and the risk of vitiligo and phototherapy effects in the Chinese Han population. Two independent samples were enrolled to form the discovery set (comprised of 1668 nonsegmental vitiligo [NSV] patients and 2542 controls) and the validation set (comprised of 745 NSV patients and 1492 controls). A total of 13 tag single nucleotide polymorphisms (SNPs) were genotyped in the samples from the discovery stage. SNPs that achieved nominal significance were validated in another independent sample set. The serum level of NR1H3 protein was assayed using enzyme-linked immunosorbent assay kits in the validation set. Genetic association analysis was carried out at allelic and genotypic levels. The therapeutic effects of significant SNPs were examined in the validation set. The SNP rs3758672 was significantly associated with NSV. The A allele was correlated with NSV risk and poorer therapeutic effects. The A allele was strongly correlated with the increased level of serum NR1H3 in both controls and patients. In summary, SNP rs3758672 in *NR1H3* was significantly associated with both disease susceptibility and individualized therapeutic effects of NSV in study participants with Han Chinese ancestry.

## Introduction

With an estimated prevalence of 0.5–2% worldwide, vitiligo is the most prevalent depigmenting disorder^1^. According to a large-scale community-based survey from six Chinese cities, the overall prevalence of vitiligo was 0.56%^2^. There are two major types: segmental and nonsegmental vitiligo^3^. The nonsegmental form is characterized by bilateral, symmetrical and frequently progressive vitiligo, and segmental vitiligo is unilateral and has a characteristic pattern of distribution^4^. Although it has less impact on patients' longevity, vitiligo can have a negative impact and psychosocial effect on patients' quality of life. Thus, effective novel therapies are urgently needed. Studies have shown that vitiligo is a complex disorder affected by multiple factors and immune-mediated melanocyte destruction leading to concert^5^. An early family-based study indicated that immediate relatives of vitiligo patients have an increased risk of vitiligo, indicating that genetic factors play key roles in the etiology of vitiligo^6^. While multiple genes have been identified, such as *PTPN22* and *NALP1,* in vitiligo^7,8^, the basic pathogenesis remains unclear.

The *NR1H3* gene is one of the many susceptibility genes that deserve attention. The *NR1H3* gene located on 11p11.2 and expressed in the skin belongs to the nuclear hormone receptor superfamily of ligand-activated transcription factors^9^. Upregulation of NR1H3 stimulates keratinocyte differentiation, proliferation and apoptosis and improves permeability barrier homeostasis. Anti-inflammatory effects and reduced inflammation are related to the activation of *NR1H3*^10^. *NR1H3* is also involved in the metabolic checkpoint that modulates cell proliferation in skin and participates in lamellar body secretion, epidermal lipid synthesis and lipid processing in the stratum corneum^11^. Moreover, *NR1H3* was found to be expressed in melanocytes^12^, and many genes related to the regulation of melanocytes were target genes of *NR1H3*^13^. More importantly, the perilesional skin of vitiligo patients had significantly higher expression of *NR1H3* than normal skin^12^. In addition, upregulated expression of *NR1H3* in perilesional skin melanocytes was significantly associated with decreased proliferation, adhesion and matrix metalloproteinases and increased apoptosis^13^. Collectively, these studies show that *NR1H3* might be a key gene in the pathogenesis of vitiligo. It has been reported that a polymorphism located at the 5′ untranslated region in NR1H3 is associated with susceptibility to vitiligo risk in the North Indian population^14^. A recent study also showed that this polymorphism and another non-synonymous variant in the exonic region of the *NR1H3* gene are associated with the risk of vitiligo in Egyptian study participants; however, the relationship between any clinical features of vitiligo and relevant genetic polymorphisms was not fully examined^15^.

Although some studies have been conducted, their conflicting results leave the relationship between the *NR1H3* gene and vitiligo risk still to be elucidated. Do the polymorphisms in the *NR1H3* gene contribute to the risk of vitiligo in Han Chinese individuals? Is serum NR1H3 protein level associated with vitiligo severity and individualized treatment effects? These are questions that need to be addressed, especially in the Han Chinese population. Given that the *NR1H3* gene is a significant option for identifying the predictive variations in the individualized prevention and treatment of vitiligo, therefore, the purpose of our study is to determine whether the genetic polymorphisms of *NR1H3* would contribute to the susceptibility and effects of treatment to NSV in Chinese Han population.

## Materials and methods

### Study subjects

**Figure 1:**
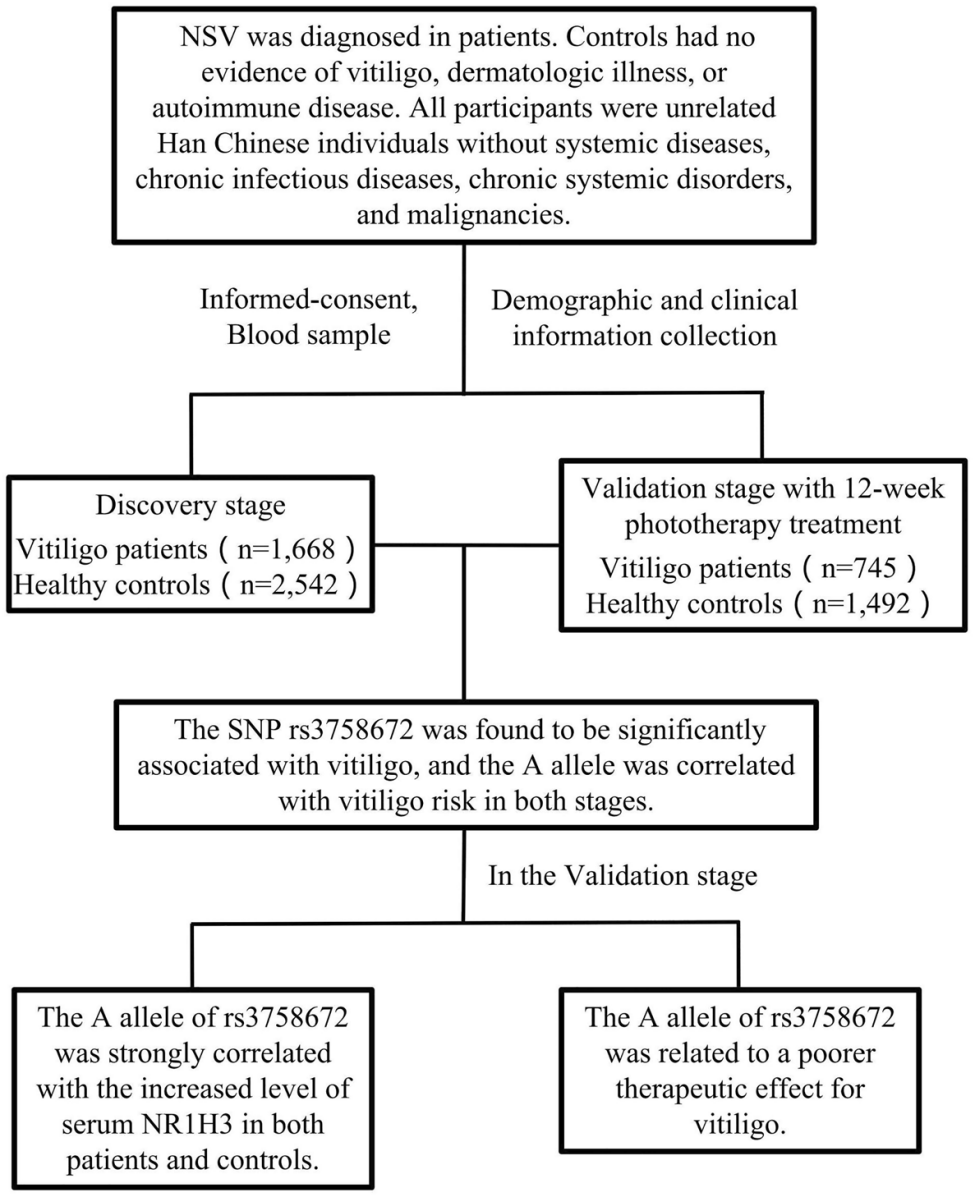
Flowchart of the study and participants’ enrollment.

As shown Fig. 1, two separate samples were included for the discovery and validation stages in this study. The discovery stage contained 1668 nonsegmental vitiligo (NSV) patients and 2542 healthy age-matched controls, and the validation set consisted of 745 NSV patients and 1492 healthy age-matched controls. All subjects were unrelated Han Chinese individuals without a history of migration within three generations and were recruited from the Department of Dermatology of the Second Affiliated Hospital of Xi’an Jiaotong University. According to the clinical assessment, Wood’s light examination, and dermoscopic evaluation, NSV was diagnosed in patients. There was no evidence of vitiligo, dermatologic illness, or autoimmune disease in any of the controls. In addition, the study excluded participants with systemic diseases, chronic infectious diseases, chronic systemic disorders, and malignancies. In addition to the general information, a complete medical history was gathered for each patient, who underwent a thorough general checkup and a rigorous dermatological examination to determine the distribution of lesions and the clinical type of patients. Disease activity was assessed based on a Vitiligo Disease Activity (VIDA) score^16^, and lower scores represent weaker activity. The Vitiligo Area Severity Index (VASI) was used to assess the severity of the disease^17^, and a high VASI score indicated high severity. The patients in the validation set received 12 weeks of phototherapy treatment (3 sessions per week) by narrowband UVB light (NB-UVB), and the VASI after treatment was extracted from their medical records. Informed consent was signed by all of the participants. In addition, 5 ml of peripheral venous blood of all participants was collected when sampling*.* This study was reviewed and approved by the Ethics Committee of Xi’an Jiaotong University, and the Declaration of Helsinki (version 2002) served as the foundation for the methods.

### SNP selection and genotyping

A total of 42 SNPs with minor allele frequency (MAF) > 0.01 and located in the *NR1H3* gene region were extracted using the 1000 genome data as a reference. Then, 13 tag SNPs were selected (r^2^ = 0.8) for genotyping in samples from the discovery stage (Supplementary Table S1). SNPs that achieved nominal significance (*P* < 0.05) in the discovery set were then genotyped in the validation set. Bonferroni corrections were applied for addressing multiple comparisons in the validation stage*.*

Peripheral blood samples extracted from study participants were utilized for genomic DNA extraction. This experiment was performed using a commercial DNA kit (Axygen Scientific, Inc., Union City, California, USA). The human DNA samples were then used for genotyping through the Sequenom MassARRAY platform. Typical MALDI-TOF results for SNP rs3758672 were showed in Supplementary Fig. S1. Replication experiments were performed on 5% of randomly selected DNA samples as a quality control procedure. Genotype calls were then released for further data analyses. The levels of serum NR1H3 were assayed using enzyme-linked immunosorbent assay (ELISA) kits (DSL Co., Ltd., Shanghai, China) in the validation set. Operations were conducted according to the kit's instructions.

### Statistical analyses

Power analyses were performed using GAS power calculator (https://csg.sph.umich.edu/abecasis/gas_power_calculator/). The parameter settings and results of the power analysis were showed in Supplementary Table S2 and Fig. S2. The Shapiro–Wilk test was performed to check the normality of the continuous variables. Analysis of variance (ANOVA) and Student's *t* test were performed for continuous data that passed normality checks. Otherwise, nonparametric methods, including the Wilcoxon rank-sum test and Kruskal–Wallis (K-W) test, were conducted. The χ^2^ test was utilized for categorical data. Hardy–Weinberg equilibrium (HWE) tests were performed in the controls as a quality control procedure. Genetic association analysis was performed at allelic and genotypic levels. Plink v1.9 was utilized for genetic association analyses^18^. The linkage disequilibrium (LD) structures of the genotyped SNPs were visualized using Haploview v4.2^19^. The relationship between significant SNPs and the therapeutic effect of phototherapy treatment in participants with NSV from the validation set was also investigated. The relationship between the serum level of NR1H3 and study participants with different genotypes of significant SNPs was also examined. The genotype-tissue expression (GTEx) database was utilized to explore the functional consequences of relevant DNA variants by measuring their effects on the gene expression level of *NR1H3*^20^.

## Results

### Characteristics and demographic information of the study participants

A total of 4210 participants comprising 1668 NSV patients and 2542 controls were enrolled in the discovery stage. The validation set was formed by 745 NSV patients and 1492 controls (Table 1). Blood samples were collected from all these study participants in both discovery and validation stages. Among the characteristic variables, only the distribution of leukotrichia was identified to be significantly different in patients (χ^2^ = 35.4, *P* < 0.01) and controls (χ^2^ = 262.4, *P* < 0.01) in both sets. The average VASI scores for patients with NSV in the validation set were 4.53 and 3.30 in the pre- and post-treatment groups, respectively.

**Table 1:** Demographic and characteristics of the study subjects.

Variables	Discovery set (N = 4210)		Statistics	*P*-value	Validation set (N = 2237)		Statistics	*P*-value
	Patients (N = 1668)	Controls (N = 2542)			Patients (N = 745)	Controls (N = 1492)		
Age, years	32.6 ± 11.5	32.5 ± 11.8	*t* = 0.12	0.91	35.7 ± 13.0	35.7 ± 13.7	*t* = − 0.03	0.98
Sex (%)								
Male	981 (59)	1494 (59)			466 (63)	933 (63)		
Female	687 (41)	1048 (41)	χ^2^ = 0.00	1.00	279 (37)	559 (37)	χ^2^ = 0	1.00
Family history (%)								
Yes	220 (13)	331 (13)			80 (11)	162 (11)		
No	1448 (87)	2211 (87)	χ^2^ = 0.01	0.91	665 (89)	1330 (89)	χ^2^ = 0.0002	0.99
Leukotrichia (%)								
Yes	262 (16)	281(11)			228 (31)	81 (5)		
No	1282 (84)	2385(89)	χ^2^ = 35.4	< 0.01	517 (69)	1411(95)	χ^2^ = 262.41	< 0.01
Residence (%)								
Rural	445 (27)	680 (27)			255 (34)	512 (34)		
Urban	1223 (73)	1862 (73)	χ^2^ = 0.0003	0.99	490 (66)	980 (66)	χ^2^ = 0.00	1.00
Onset (%)								
Sudden	382 (23)	–	–	–	221 (30)	–	–	–
Gradual	1286 (77)	–	–	–	524 (70)	–	–	–
Disease type (%)								
Generalized	643 (39)	–	–	–	252 (34)	–	–	–
Acrofacial	574 (34)	–	–	–	288 (39)	–	–	–
Mucosal	262 (16)	–	–	–	126 (17)	–	–	–
Universal	189 (11)	–	–	–	79 (10)	–	–	–
NSV disease activity (%)								
0	396 (24)	–	–	–	–	–	–	–
1	393 (24)	–	–	–	237 (32)	–	–	–
2	318 (19)	–	–	–	184 (25)	–	–	–
3	306 (18)	–	–	–	144 (19)	–	–	–
4	255 (15)	–	–	–	180 (24)	–	–	–
NSV Area Severity Index								
Pre-treatment	3.76 ± 1.85	–	–	–	4.53 ± 1.85	–	–	–
Post-treatment		–	–	–	3.30 ± 1.73	–	–	–
Serum level of NRIH3, μg/ml		–	–	–	5.18 ± 1.12	2.73 ± 0.51	–	–

Continuous variables were presented by mean ± standard deviation.

### Genetic association between genetic polymorphisms and NSV susceptibility

All the SNPs genotyped in the discovery set were in Hardy–Weinberg equilibrium (Supplementary Table S1). Three SNPs, rs4320917 (χ^2^ = 4.23, *P* = 0.04), rs3758672 (χ^2^ = 21.95, *P* = 2.81 × 10^–6^) and rs2279238 (χ^2^ = 4.14, *P* = 0.04), achieved nominal significance in the discovery set (Table 2 and Supplementary Table S3). In the validation set, only rs3758672 was found to be significant (χ^2^ = 13.43, *P* = 2.48 × 10^–4^). The A allele at rs3758672 was significantly linked to higher susceptibility to NSV (OR [95% CI] = 1.25 [1.14–1.38]). The results of genotypic analysis further presented the dose-dependence pattern of the genetic effects. Compared to the study participants with the GG genotype (reference group), the ORs of individuals with the AA and AG genotypes were 1.61 (1.18–2.21) and 1.33 (1.10–1.60), respectively. The LD structures of the genotyped SNPs in the discovery and validation sets are shown in Supplementary Figs. S3 and S4. Additionally, no association was detected between genotypes of rs3758672 and clinical features of NSV in the patients from both the discovery and validation sets (Supplementary Table S4).

**Table 2:** Results of genetic association analyses for SNPs genotyped in both discovery and validation sets.

SNP	Tests	Group	Discovery set (N = 4210)		OR (95%CI)	χ^2^	*P*-value	Validation set (N = 2237)		OR(95%CI)	χ^2^	*P*-value
			Patients (N = 1668)	Controls (N = 2542)				Patients (N = 745)	Controls (N = 1492)			
rs4320917	Genotypic	TT	230 (14)	320 (13)	1.18 (0.97–1.44)			104 (14)	187 (13)	1.25 (0.95–1.65)		
		CT	784 (47)	1144 (45)	1.13 (0.99–1.29)			358 (48)	668 (45)	1.21 (1.00–1.46)		
		CC	654 (39)	1078 (42)	ref	4.49	0.11	283 (38)	637 (42)	ref	4.62	0.10
	Allelic	T	1244 (37)	1784 (35)	1.10 (1.00–1.20)			566 (38)	1042 (35)	1.14 (1.00–1.30)		
		C	2092 (63)	3300 (65)	ref	4.23	0.04	924 (62)	1942 (65)	ref	4.06	0.04
	HWE *P*-value	–	0.88	0.54	–	–	–	0.64	0.57	–	–	–
rs3758672	Genotypic	AA	180 (11)	200 (8)	1.58 (1.27–1.98)			79 (11)	116 (8)	1.61 (1.18–2.21)		
		AG	764 (46)	1068 (42)	1.20 (1.05–1.36)			346 (46)	618 (41)	1.33 (1.10–1.60)		
		GG	724 (43)	1274 (50)	ref	22.42	1.35 × 10^–5^	320 (43)	758 (51)	ref	13.83	9.94 × 10^–4^
	Allelic	A	1124 (34)	1468 (29)	1.25 (1.14–1.38)			504 (34)	850 (28)	1.28 (1.12–1.47)		
		G	2212 (66)	3616 (71)	ref	21.95	2.81 × 10^–6^	986 (66)	2134 (72)	ref	13.43	2.48 × 10^–4^
	HWE *P*-value	–	0.32	0.27	–	–	–	0.33	0.57	–	–	–
rs2279238	Genotypic	CC	253 (15)	445 (18)	0.82 (0.68–0.98)			113 (15)	258 (17)	0.79 (0.61–1.03)		
		CT	801 (48)	1215 (48)	0.95 (0.83–1.09)			356 (48)	734 (49)	0.88 (0.72–1.07)		
		TT	614 (37)	882 (34)	ref	4.60	0.10	276 (37)	500 (34)	ref	3.35	0.19
	Allelic	C	1307 (39)	2105 (41)	0.91 (0.83–1.00)			582 (39)	1250 (42)	0.89 (0.78–1.01)		
		T	2029 (61)	2979 (59)	ref	4.14	0.04	908 (61)	1734 (58)	ref	3.29	0.07
	HWE *P*-value	–	0.80	0.46	–	–	–	0.94	0.71	–	–	–

HWE: *P* value of the Hardy–Weinberg equilibrium.

The threshold of P value is 0.05/3≈0.017.

### Association between rs3758672 and therapeutic effect for NSV

Phototherapy treatment was generally effective for NSV patients in the validation set (Supplementary Fig. S5). The reduction in the VASI score after 12 weeks of phototherapy treatment was not normally distributed (Supplementary Fig. S6), and nonparametric statistical methods were applied to test the relationship between relevant variables and the reduction in the VASI. The reduction in VASI was significantly associated with genotypes of rs3758672 (Kruskal‒Wallis χ^2^ = 16.19, *P* = 3.06 × 10^–4^) and NSV disease activity (Kruskal‒Wallis χ^2^ = 15.66, *P* = 1.33 × 10^–3^) in patients in the replication set (Table 3). The A allele of rs3758672 was related to a poorer therapeutic effect for NSV. This dose-dependent pattern was not changed after stratifying the patients by NSV disease activity (Supplementary Fig. S7).

**Table 3:** Relationship between reduction of VASI and baseline variables in patients of the validation set.

Variables	Reduction of VASI	Statistics	*P*-Value
Age	–	Pearson's r = 0.02	0.54
Genotypes of rs3758672 (N)			
AA (79)	1.04 (0.47–1.54)		
AG (346)	1.33 (0.78–1.67)		
GG (320)	1.47 (0.89–1.77)	Kruskal–Wallis χ^2^ = 16.19	3.06 × 10^–4^
Sex (N)			
Male (466)	1.37 (0.80–1.72)		
Female (279)	1.41 (0.78–1.75)	W = 66,072	0.71
Residence (N)			
Rural (255)	1.36 (0.80–1.72)		
Urban (490)	1.37 (0.79–1.73)	W = 63,421	0.73
Onset (N)			
Sudden (221)	1.39 (0.88–1.74)		
Gradual (521)	1.36 (0.77–1.72)	W = 60,005	0.43
Leukotrichia (N)			
Yes (228)	1.44 (0.84–1.74)		
No (517)	1.34 (0.78–1.72)	W = 62,410	0.20
Disease type (N)			
Generalized (252)	1.36 (0.75–1.70)		
Acrofacial (288)	1.40 (0.81–1.75)		
Mucosal (126)	1.31 (0.90–1.69)		
Universal (79)	1.43 (0.82–1.77)	Kruskal–Wallis χ^2^ = 2.64	0.45
NSV disease activity (N)			
1 (237)	1.29 (0.60–1.64)		
2 (184)	1.47 (0.99–1.78)		
3 (144)	1.29 (0.80–1.67)		
4 (180)	1.47 (1.01–1.78)	Kruskal–Wallis χ^2^ = 15.66	1.33 × 10^–3^

VASI: Vitiligo Area Severity Index. Continuous variables were presented by median value with interquartile range.

The *P* values for Hardy–Weinberg equilibrium tests of rs3758672 in the validation set were 0.33 and 0.57 for cases and controls, respectively.

### Functional consequences of rs3758672

**Figure 2:**
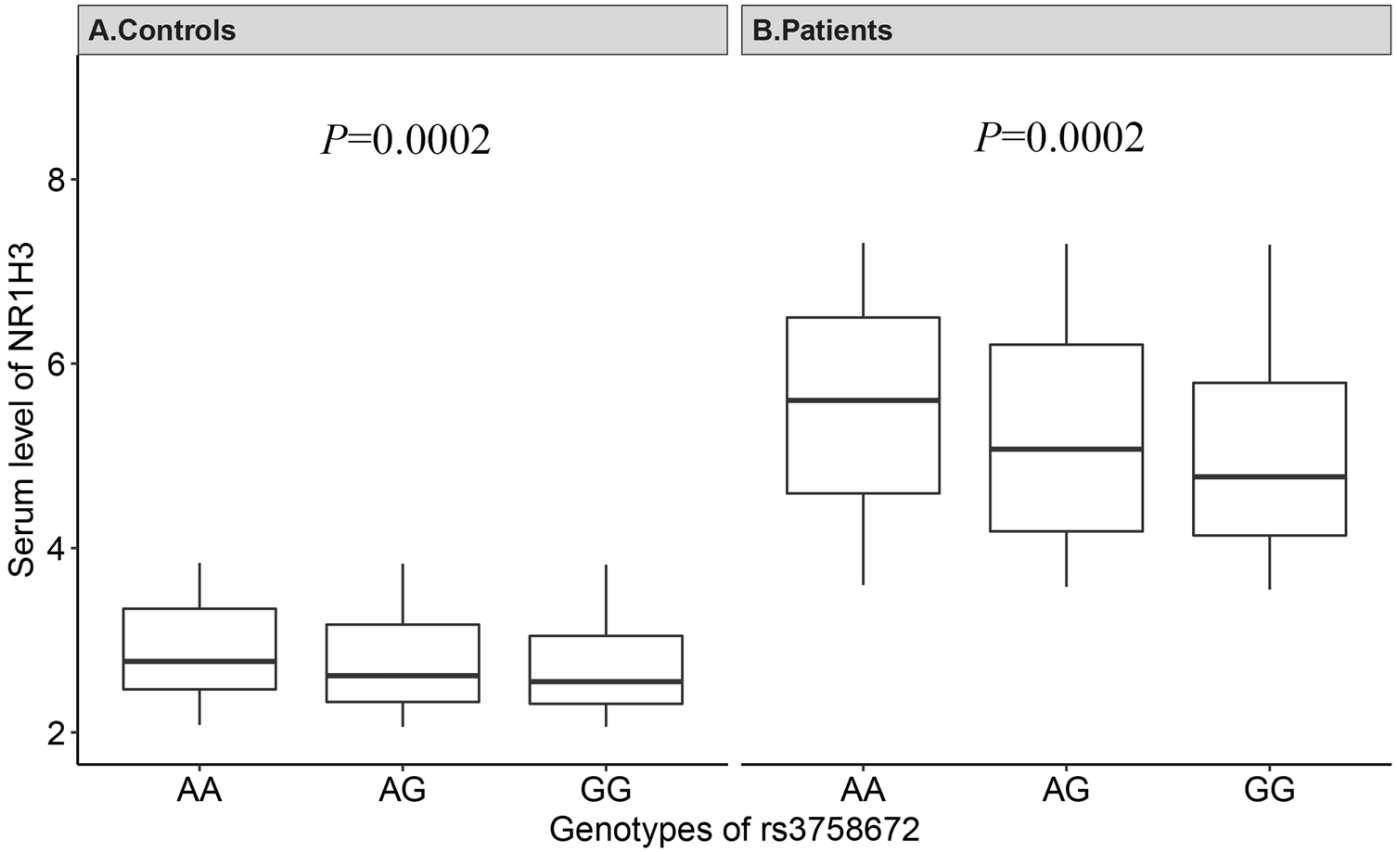
Serum levels of NR1H3 for participants with different genotypes of SNP rs3758672. (**A**) Controls, (**B**) patients with NSV. Kruskal–Wallis (K-W) tests were conducted.

**Figure 3:**
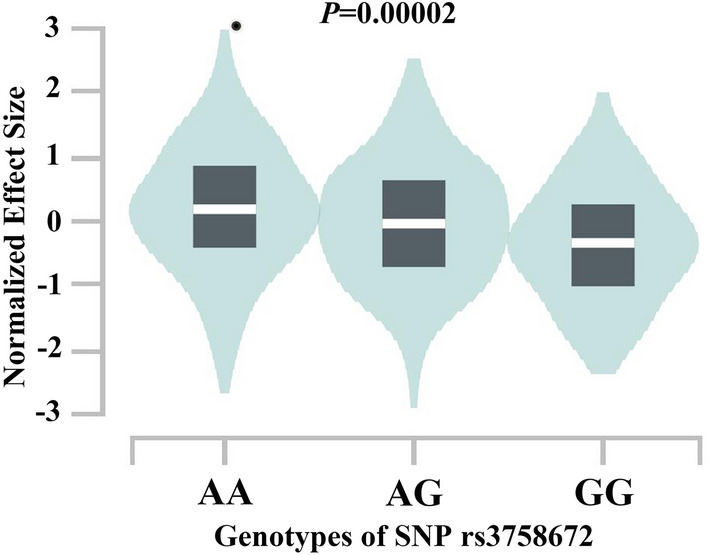
Expression levels of *NR1H3* in human blood tissues grouped by different genotypes of rs3758672.

The relationship between the serum level of NR1H3 and genotypes of rs3758672 was examined and is presented in Fig. 2. In both groups of controls (Fig. 2A, K-W χ^2^ = 16.44, *P* = 0.0002) and patients with NSV (Fig. 2B, K-W χ^2^ = 16.25, *P* = 0.0002), the A allele was found to be significantly correlated with a higher serum level of NR1H3. This pattern was in accordance with the *NR1H3* expression level in human whole blood tissue using data from GTEx (Fig. 3). In addition, in samples of whole blood, the A allele was substantially correlated with greater levels of *NR1H3* expression.

## Discussion

Nuclear receptor subfamily 1 Group H member 3 encodes a protein belonging to the NR1 subfamily of the nuclear receptor superfamily. Previous genome-wide association studies (GWAS) have linked the genetic polymorphisms of the *NR1H3 gene* to a variety of human diseases and phenotypes, including blood cholesterol levels, blood pressure and hypertension, and metabolic syndrome^21^. However, no GWAS has reported a significant association between the *NR1H3* gene and NSV and its related phenotypes. Nevertheless, two candidate gene-based studies in Indian and Egyptian populations have linked *NR1H3* genetic polymorphisms to susceptibility to vitiligo^14,15^. Both SNPs rs11039155 and rs2279238 were significant hits in these studies. In the present work, only SNP rs2279238 was selected for genotyping in the discovery set but was not identified to be significant. Instead, we identified rs3758672 to be associated with susceptibility to vitiligo in a large study sample with Chinese Han ancestry based on a two-stage design. The differences in association signals between the current study and these previous studies might be explained by the genetic heterogeneity of vitiligo. The differences in the linkage disequilibrium patterns of different populations might be the origin of the discordance. Moreover, this allelic heterogeneity could also indicate that all of these reported significant hits might just be surrogates for some undiscovered DNA variants that were not analyzed in any of these studies since it is quite unlikely to identify two independent association signals with true effect on disease susceptibility.

To systematically evaluate the relationship between polymorphisms of the *NR1H3* gene and susceptibility to NSV risk, multiple lines of evidence were investigated and gathered in the present study. In addition to the direct association signal identified through a traditional genetic association analysis, the genetic association between the therapeutic effect for NSV and genotypes of rs3758672 was also explored. A recent population-based study evaluated the relationship between genetic polymorphisms of the nuclear vitamin D receptor (VDR) gene and vitiligo patients’ response to narrowband UVB phototherapy in a sample of Egyptians^22^. Compared to this recent study, we identified another independent genetic predictor of the patients' response to phototherapy. Although it is out of the scope of the work to determine the potential causes of this genetic effect on the treatment of NSV, it is still worthy of general discussion. First, this effect could not be explained by the severity of NSV because according to the results of the stratification analysis, this effect was in the same pattern in each subgroup of NSV patients. We hypothesize that this effect might originate from the deep pathogenesis of NSV, and future studies are required to explore the mechanisms of this effect.

Evidence of the functional consequences for the genetic association signals was also investigated at both the gene expression and protein levels. The A allele of rs3758672 was associated with both the expression level of *NR1H3* in human blood tissue and the serum level of NR1H3. Interestingly, a previous study showed that keratinocyte *NR1H3* had a significantly higher expression in lesional skin than in control skin^23^, and *NR1H3* upregulation was associated with keratinocyte damage^23^. In addition, a couple of previous studies have revealed higher expression levels of NR1H3 in perilesional skin compared to the normal skin of vitiligo patients^24,25^. An increase in the expression of NR1H3 would reduce or inhibits the expression of matrix metalloproteinases (MMPs). In perilesional vitiligo skin, a decrease in MMPs would inhibit the migration or replacement of melanocytes from hair outer root sheath melanoblasts. Based on the above results, we could form a hypothesis to illustrate the potential mechanisms of the genetic effects of *NR1H3* on susceptibility to NSV by synthesizing all of this evidence. The A allele at SNP rs3758672 is correlated with higher gene expression and protein levels of *NR1H3,* and this in turn might cause lesions in the skin tissue and increase susceptibility to NSV. This same allele is related to increased susceptibility to NSV and is related to poorer therapeutic effects for NSV patients. Notably, this would contradict the anti-inflammatory effects of NR1H3. Although pharmacological activation of LXR has been shown to improve the severity of inflammatory responses, similar results have not been reported for vitiligo. Different pathways play roles in the pathogenesis of complex diseases, and the same molecule does not necessarily play the same role in different pathways. Considering the complexity of the pathogenesis of vitiligo and the insufficiency of functional research on LXR-α (the protein encoded by NR1H3 gene), follow-up research on the relationship between the NR1H3 gene and the pathogenesis of vitiligo is still needed.

This study suffers from several limitations. First, the target tissue of NSV is human skin. However, in this study, we only tested the NR1H3 protein level in the serum. This might not accurately reflect the NR1H3 protein level in human skin. In addition, data on the gene expression level of *NR1H3* measured in human skin were extracted from a publicly available database. The characteristic information from tissue donors is largely missed. Thus, the gene expression pattern of *NR1H3* reported in data from the GTEx database might be different from the gene expression patterns in NSV patients. In addition, since information on the age of onset of the NSV patients were not collected, no further stratification analysis could be conducted to examine the potential SNP effects in different subtypes of NSV patients. Finally, as a candidate gene-based study, only a couple of common DNA variants were genotyped in the discovery set of this study. The genetic information coverage might not be enough for unraveling the genetic contribution to the susceptibility of NSV. A large number of DNA variants, both common and rare or low-frequency, were not examined in the current work. Recent sequencing-based studies have indicated that DNA variants with low MAF might also contribute significantly to the risk of human disorders^26^. Therefore, given the molecular mechanisms of complex diseases involving multidimensional molecular interactions^27–32^, targeted sequencing-based integrative studies are needed to examine the genetic architecture of *NR1H3*.

## Conclusion

In summary, the SNP rs3758672 in *NR1H3* gene was significantly associated with both NSV susceptibility and individualized therapeutic effects of NSV in Chinese Han population. The findings of this study elucidate the role of *NR1H3* gene in NSV pathogenesis mechanisms and individualized treatment response to phototherapy and, in turn, shed light on precision medicine and the individualized treatment of NSV in the future.

## Supplementary Information

Supplementary Information.

## Data Availability

The datasets used and/or analyzed during the current study available from the corresponding author on reasonable request.
