# Supplementary material for: Contributions of NR1H3 genetic polymorphisms to susceptibility and effects of narrowband UVB phototherapy to nonsegmental vitiligo
Source: Sci Rep. 2023 Feb 28;13:3384. doi: 10.1038/s41598-023-30047-7 (PMC9974977; doi:10.1038/s41598-023-30047-7)
Supplement: Supplementary file 1 — Supplementary Information. [file 41598_2023_30047_MOESM1_ESM.docx]

***Title:*** Contributions of *NR1H3* genetic polymorphisms to susceptibility and effects of narrowband UVB phototherapy to nonsegmental vitiligo

***Author names and affiliations***: Meifeng Xu ^a^, Qiuyu Xu ^b^, Yan Liu ^a^, Xiaoli Li ^a^, Mei Wang ^a^, Wei Dong ^c^, Yuning Song ^d^, Shengxiang Xiao ^a*^

^a^ Department of Dermatology, the Second Affiliated Hospital of Xi'an Jiaotong University, Xi’an, Shaanxi, China;

^b^ School of Medicine and Forensics, Xi'an Jiaotong University Health Science Center, Xi'an, Shaanxi, China;

^c^ Department of Laboratory Medicine, the Second Affiliated Hospital of Xi'an Jiaotong University, Xi'an, Shaanxi, China;

^d^ School of Life Science and Technology, Xi'an Jiaotong University, Xi'an, Shaanxi, China.

Meifeng Xu and Qiuyu Xu contributed equally to the work.

***Corresponding author***:

Shengxiang Xiao, Department of Dermatology, the Second Affiliated Hospital of Xi'an Jiaotong University, 157 Xiwu Road, Xincheng District, Xi'an, 710004, China.

Tel: 86-29-87679329; Fax: 86-29-87679329; E-mail: shxaxiaoxjtu@163.com

Supplementary Table S1. Genetic information of the SNPs selected for genotyping in the discovery set.

| Chromosome | Position | SNP | Function | A1 | A2 | MAF | HWE_controls | HWE_cases |
| --- | --- | --- | --- | --- | --- | --- | --- | --- |
| 11 | 47255124 | rs11039149 | intron | G | A | 0.05 | 0.33 | 0.80 |
| 11 | 47258669 | rs12226869 | intron | G | C | 0.03 | 0.18 | 0.43 |
| 11 | 47258794 | rs138395745 | intron | C | T | 0.04 | 0.41 | 0.18 |
| 11 | 47264356 | rs149601947 | intron | T | C | 0.03 | 0.18 | 0.45 |
| 11 | 47262333 | rs181443960 | intron | T | C | 0.04 | 0.80 | 0.41 |
| 11 | 47260473 | rs2279238 | coding-synon | C | T | 0.41 | 0.46 | 0.80 |
| 11 | 47256866 | rs3758672 | intron | A | G | 0.31 | 0.27 | 0.32 |
| 11 | 47255543 | rs4320917 | intron | T | C | 0.36 | 0.54 | 0.88 |
| 11 | 47258377 | rs61896015 | intron | A | C | 0.12 | 0.70 | 0.72 |
| 11 | 47258214 | rs74924988 | intron | A | G | 0.07 | 0.89 | 0.58 |
| 11 | 47255307 | rs76002224 | intron | C | G | 0.08 | 0.90 | 0.50 |
| 11 | 47262058 | rs77663386 | intron | T | C | 0.03 | 0.38 | 0.23 |
| 11 | 47267848 | rs77782011 | intron | A | G | 0.03 | 0.38 | 0.23 |

A1: minor allele; A2: major allele; MAF: minor allele frequency. HWE_controls: *P* values for Hardy-Weinberg equilibrium tests conducted in controls. HWE_cases: *P* values for Hardy-Weinberg equilibrium tests conducted in cases.

Supplementary Table S2. Parameters utilized for the power calculations.

| Parameters | Values | Note |
| --- | --- | --- |
| No. of Cases | 2413 |  |
| No. of Controls | 4034 |  |
| Significance level | 0.004 | 0.05/13 |
| Prevalence of the disease | 0.56% | PMID: 22859054. |
| Disease allele frequency | 0.12 | Average of the MAF of the 13 tag SNPs |

Supplementary Table S3. Full results of the genetic association analyses for SNPs genotyped in the discovery set.

| CHR | SNP | A1 | A2 | TEST | AFF | UNAFF | χ^2^ | DF | *P* |
| --- | --- | --- | --- | --- | --- | --- | --- | --- | --- |
| 11 | rs11039149 | G | A | GENO | 5/163/1500 | 10/254/2278 | 0.31 | 2 | 0.86 |
|  |  |  |  | ALLELIC | 173/3163 | 274/4810 | 0.17 | 1 | 0.68 |
| 11 | rs76002224 | C | G | GENO | 8/247/1413 | 16/386/2140 | 0.52 | 2 | 0.77 |
|  |  |  |  | ALLELIC | 263/3073 | 418/4666 | 0.31 | 1 | 0.58 |
| 11 | rs4320917 | T | C | GENO | 230/784/654 | 320/1144/1078 | 4.49 | 2 | 0.11 |
|  |  |  |  | ALLELIC | 1244/2092 | 1784/3300 | 4.23 | 1 | 0.04 |
| 11 | rs3758672 | A | G | GENO | 180/764/724 | 200/1068/1274 | 22.42 | 2 | 0.00 |
|  |  |  |  | ALLELIC | 1124/2212 | 1468/3616 | 21.95 | 1 | 0.00 |
| 11 | rs74924988 | A | G | GENO | 10/220/1438 | 13/351/2178 | 0.46 | 2 | 0.79 |
|  |  |  |  | ALLELIC | 240/3096 | 377/4707 | 0.15 | 1 | 0.70 |
| 11 | rs61896015 | A | C | GENO | 20/344/1304 | 38/529/1975 | 0.69 | 2 | 0.71 |
|  |  |  |  | ALLELIC | 384/2952 | 605/4479 | 0.29 | 1 | 0.59 |
| 11 | rs12226869 | G | C | GENO | 3/107/1558 | 5/153/2384 | NA | NA | NA |
|  |  |  |  | ALLELIC | 113/3223 | 163/4921 | 0.21 | 1 | 0.65 |
| 11 | rs138395745 | C | T | GENO | 5/123/1540 | 5/183/2354 | 0.50 | 2 | 0.78 |
|  |  |  |  | ALLELIC | 133/3203 | 193/4891 | 0.20 | 1 | 0.66 |
| 11 | rs2279238 | C | T | GENO | 253/801/614 | 445/1215/882 | 4.60 | 2 | 0.10 |
|  |  |  |  | ALLELIC | 1307/2029 | 2105/2979 | 4.14 | 1 | 0.04 |
| 11 | rs77663386 | T | C | GENO | 3/100/1565 | 5/173/2364 | NA | NA | NA |
|  |  |  |  | ALLELIC | 106/3230 | 183/4901 | 1.08 | 1 | 0.30 |
| 11 | rs181443960 | T | C | GENO | 5/147/1516 | 5/203/2334 | 1.37 | 2 | 0.50 |
|  |  |  |  | ALLELIC | 157/3179 | 213/4871 | 1.28 | 1 | 0.26 |
| 11 | rs149601947 | T | C | GENO | 3/110/1555 | 5/153/2384 | NA | NA | NA |
|  |  |  |  | ALLELIC | 116/3220 | 163/4921 | 0.46 | 1 | 0.50 |
| 11 | rs77782011 | A | G | GENO | 3/100/1565 | 5/173/2364 | NA | NA | NA |
|  |  |  |  | ALLELIC | 106/3230 | 183/4901 | 1.08 | 1 | 0.30 |

CHR: chromosome; A1: minor allele; A2: major allele; AFF: patients with vitiligo; UNAFF: controls; DF: degree of freedome.

Supplementary Table S4. Relationship between genotypes of rs3758672 and clinical features of vitiligo in the patients from both discovery and validation set.

| Variables | Genotypes of SNP rs3758672 | | | χ^2^ | *P*-Value |
| --- | --- | --- | --- | --- | --- |
|  | AA (N= 259) | AG (N=1110) | GG (N=1,044) |  |  |
| Leukotrichia (%) |  |  |  |  |  |
| *Yes* | 59 (23) | 284 (26) | 271 (26) |  |  |
| *No* | 200 (77) | 826 (74) | 773 (74) | 1.13 | 0.57 |
| Onset (%) |  |  |  |  |  |
| *Sudden* | 69 (27) | 279 (25) | 255 (24) |  |  |
| *Gradual* | 190 (73) | 831 (75) | 789 (76) | 0.57 | 0.75 |
| Disease Type (%) |  |  |  |  |  |
| *Generalized* | 104 (40) | 419 (38) | 372 (36) |  |  |
| *Acrofacial* | 85 (33) | 384 (35) | 393 (38) |  |  |
| *Mucosal* | 39 (15) | 180 (16) | 169 (16) |  |  |
| *Universal* | 31 (12) | 127 (11) | 110 (10) | 4.25 | 0.64 |
| Vitiligo Disease Activity (%) | |  |  |  |  |
| *0* | 37 (14) | 201 (18) | 158 (15) |  |  |
| *1* | 72 (28) | 267 (24) | 291 (28) |  |  |
| *2* | 69 (27) | 230 (21) | 203 (20) |  |  |
| *3* | 44 (17) | 207 (19) | 199 (19) |  |  |
| *4* | 37 (14) | 205 (18) | 193 (18) | 14.93 | 0.06 |

**Supplementary Figures**


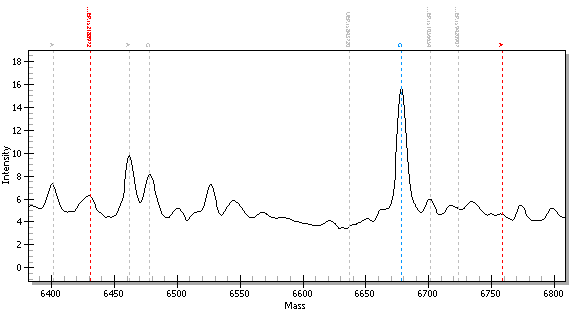
a


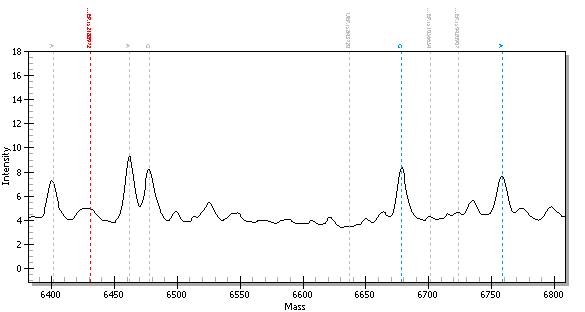
b


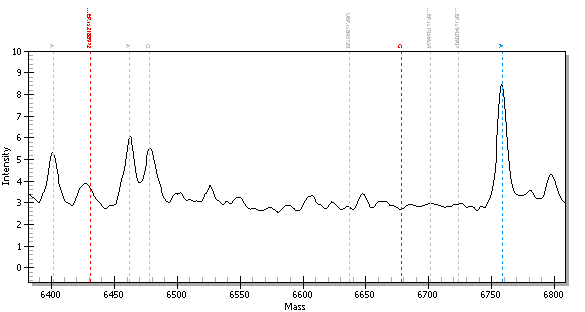
c

Supplementary Figure S1. Typical MALDI-TOF figures for GG(a), GA(b), AA(c) genotype of rs3758672.


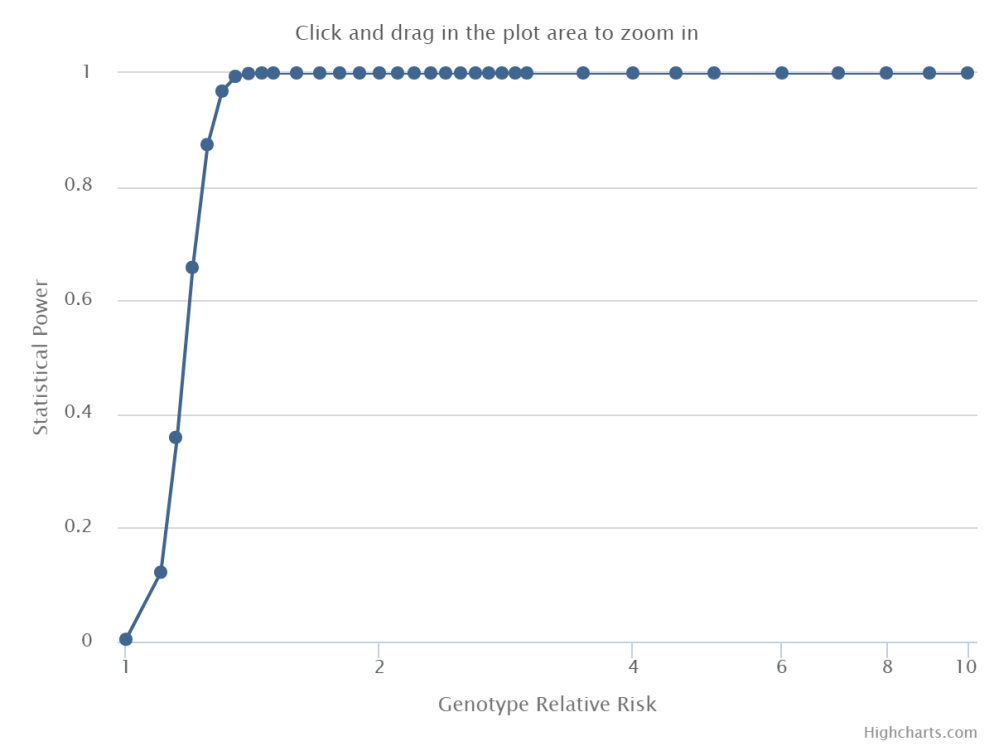


Supplementary Figure S2. Results of the power analysis.


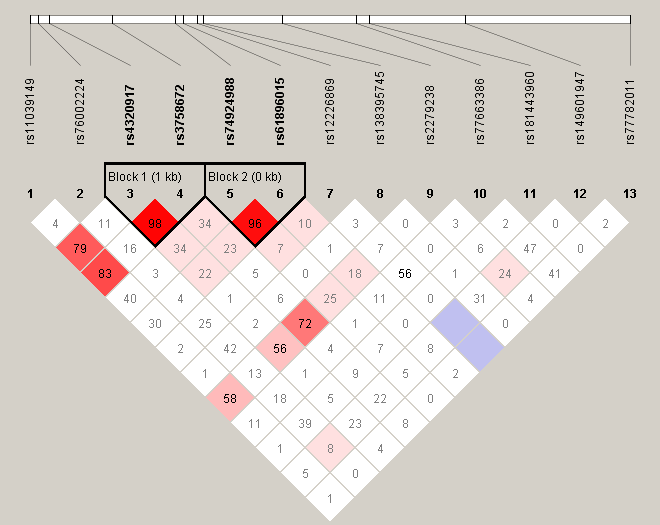


Supplementary Figure S3. LD structure of the 13 genotyped SNPs in the discovery set. Values of D' were presented in each cell.


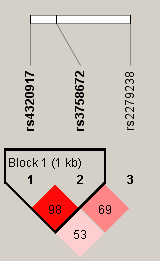


Supplementary Figure S4. LD structure of the 3 genotyped SNPs in the validation set. Values of D' were presented in each cell.


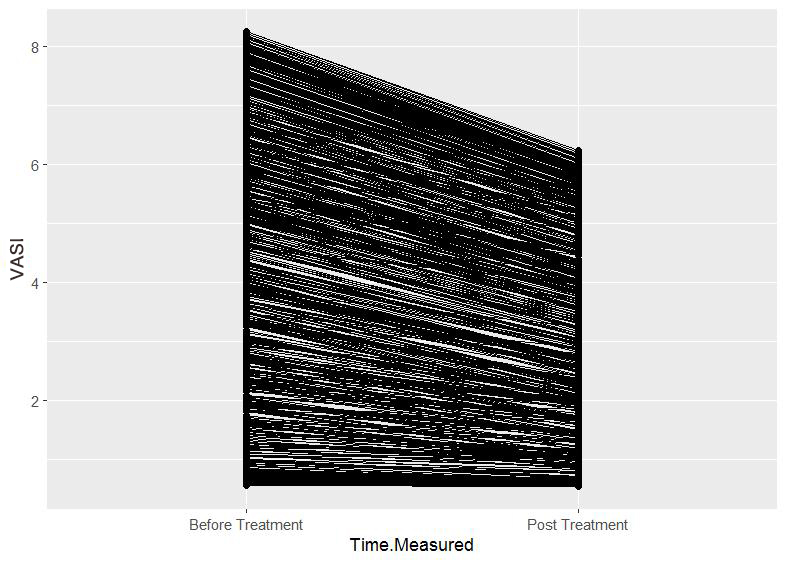


Supplementary Figure S5. Changes of the VASI scores for patients in the validation set.


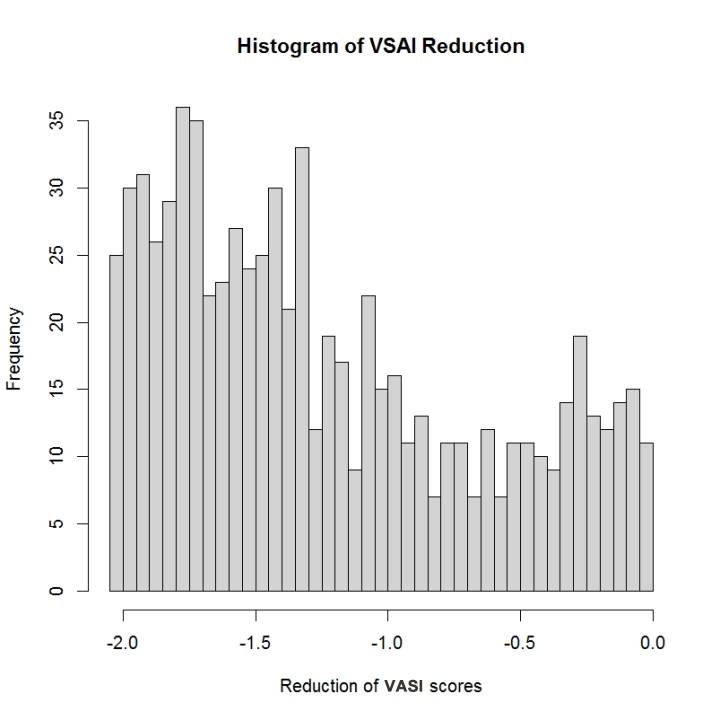


Supplemental Figure S6. Histogram of VASI reduction in the validation set.


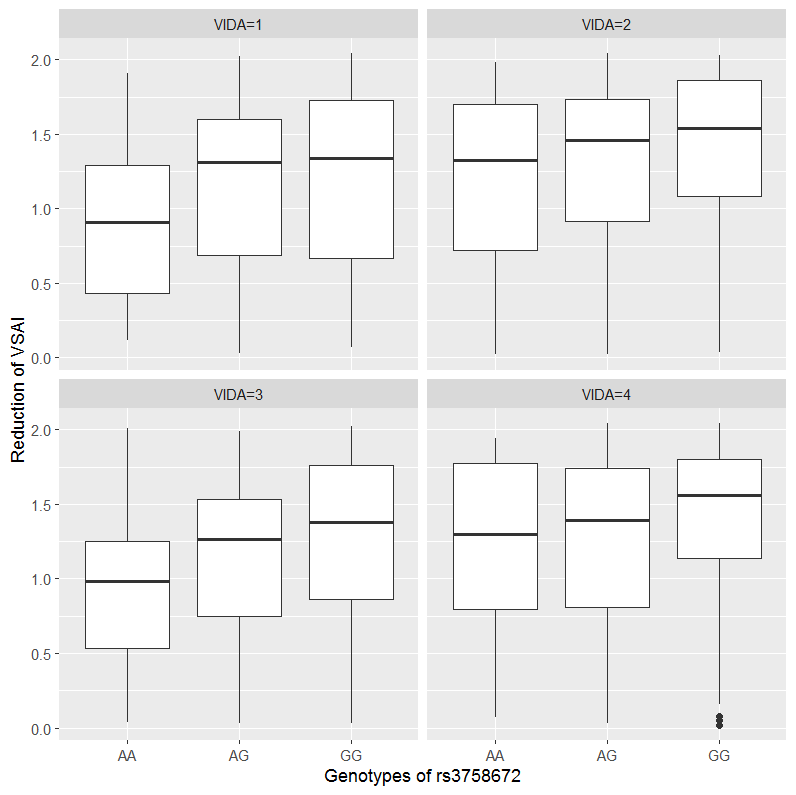


Supplementary Figure S7. Relationship between genotypes of rs3758627 and the reduction of VASI in patients of validation set stratified by VIDA.
